# The Mediating Role of Self-Efficacy in the Relationship between Approach Motivational System and Sports Success among Elite Speed Skating Athletes and Physical Education Students

**DOI:** 10.3390/ijerph19052899

**Published:** 2022-03-02

**Authors:** Aleksandra M. Rogowska, Rafał Tataruch, Konrad Niedźwiecki, Bożena Wojciechowska-Maszkowska

**Affiliations:** 1 Institute of Psychology, University of Opole, 45-052 Opole, Poland; 2 Faculty of Physical Education and Physiotherapy, Opole University of Technology, 45-758 Opole, Poland; r.tataruch@po.edu.pl (R.T.); b.wojciechowska-maszkowska@po.edu.pl (B.W.-M.); 3 Polish Speed Skating Association, 01-515 Warszawa, Poland; k.niedzwiedzki@pzls.pl

**Keywords:** approach and avoidance temperament, elite athletes, physical education, Reinforcement Sensitivity Theory (RST), self-efficacy, speed skating, sports success

## Abstract

Background: While the association between self-efficacy and sports success has been well established in previous studies, little is known regarding whether the basic approach motivation system contributes to this relationship in athletes. The study examines associations between self-reported temperamental approach disposition, self-efficacy, and predispositions to sports success in athletes. Methods: A cross-sectional study was performed between August 3 and 30 November 2020. The participants were 156 athletes, aged 16–34 years (*M* = 21.57, *SD* = 3.58, 41.67% women), in two groups: 54 elite athletes in speed skating (EASS) and 102 physical education students (PES). The online survey consisted of the Reinforced Sensitivity Questionnaire (RSQ), General Self-Efficacy Scale (GSES), and Sports Success Scale (SSS). Results: There were no differences in self-efficacy and sports success in terms of gender, sports discipline, and level of competitions. The Behavioral Activation System (BAS) results were lower in the EASS sample compared with in the PES group. Self-efficacy plays a mediating role in the relationships between BAS and sports success among athletes, with sport discipline as a moderator between BAS and self-efficacy. Sports success in speed skating relies strongly on BAS, while a weak link has been found in other sports disciplines. Conclusions: BAS is directly correlated to sports success and indirectly related through self-efficacy. Mental training should be focused on maintaining self-efficacy and reward motivation in athletes to increase their sports success.

## 1. Introduction

### 1.1. Sports Success and Self-Efficacy

According to Bandura [1,2], self-efficacy can be understood as belief in one’s abilities or as a kind of expectation or assessment of one’s own ability to cope with a given situation concerning skills and circumstances. Self-efficacy is related to self-regulation and is understood as controlling and modulating one’s behavior to achieve goals. Research indicates that people who believe in their effectiveness can outperform those who misjudge their coping skills [1]. High levels of self-efficacy are associated positively with academic success [3,4], maintaining physical activity [5,6,7,8,9,10,11], and better sports performance [11,12,13,14,15,16,17,18,19,20,21,22,23].

The meta-analysis of 45 studies showed a moderate correlation (*r* = 0.38) between self-efficacy and sports performance [11]. Research indicated that high self-efficacy positively influences physical activity enjoyment [8] and can facilitate the effectiveness of imagery used by athletes [22]. People with a high sense of self-efficacy demonstrate more positive affect and lower perceived exertion during physical exercise [10], and they endure longer, put in more effort, and tend to undertake more complex tasks, compared to those who score low in self-efficacy [5].

Self-efficacy is related to sports levels of competition. Among elite artistic roller skaters, those who successfully won a medal at competition reported higher self-efficacy level than skaters who did not [13]. Volleyball and basketball experts scored higher in self-efficacy than either non-experts or novices [15,17]. Furthermore, basketball experts outperformed novices in choosing technique-oriented strategies, formulating specific goals and better strategy attribution [15].

Furthermore, sports experts (i.e., athletes with many years of deliberate practice relating to intense involvement in structured and individualized training sessions, skill goal setting, and self-monitoring of sports performance), and more successful athletes scored higher in self-efficacy than novices (i.e., athletes who were starting training in a given sports discipline) and those who performed worse [13,15,17]. Self-efficacy is inversely related to fear of success [12].

Recent research [24] showed that gender may play a role in the relationship between self-efficacy and sports performance. For example, collective self-efficacy in rowing athletes (i.e., not individual self-efficacy, but self-efficacy related to the success of a rowing team) was significantly related to sports performance in men but not among women. Furthermore, in this research, male athletes presented a lower self-efficacy than females, contrary to other studies showing higher self-efficacy in men than in women [18,19,25,26,27,28,29]. Perceptions of gender stereotypes about male or female sports or tasks can affect self-efficacy in terms of physical activity, exercise, and athletic performance [24,25,30,31].

### 1.2. Sports Success and BIS-BAS

The Reinforcement Sensitivity Theory (RST) postulates that the approach–avoidance motivational system is fundamental for explaining individual differences in behavior and emotions in everyday life [32,33,34]. Three independent neuropsychological systems can regulate behavior based on sensitivity to different kinds of reinforcement: a behavioral approach system (BAS), a behavioral inhibition system (BIS), and a fight–flight–freeze system (FFFS). BAS is activated by appetitive and goal-oriented stimuli and is sensitive to reward signals related to positive emotions, such as excitement, hope, and happiness.

BIS is activated by conflicting stimuli (activation FFFS and BAS simultaneously by “goal conflict”) and is sensitive to punishment, non-reward, and novelty signals. Aversive stimuli activate FFFS and regulate behavior in response to unconditioned punishment. BIS and FFFS are related to aversive motivation, which controls negative emotions, fear, and anxiety. Individual differences in the sensitivity of these three systems can be considered temperamental traits, which are the basis for further personality development.

Goals and motives can predict performance to approaching success and avoiding failure [35], which is determined by the socialization process. The approach and avoidance temperamental traits are innate dispositions that contribute to developing achievement motives and regulating behavior. People with a performance approach tend to perform better compared to others. Elliot and Thrash [36] found a positive association between approach goals (mastery and performance) and the BAS, while the BIS was related to avoidance goals. Research showed that the BAS was associated with high academic achievement [37] and career exploration among university students [38].

The approach motivational system (BAS) is also related to participation in physical activity (PA) [39,40,41] and high achievement in sports performance [42]. Furthermore, Gable et al. [43] demonstrated that people with dispositional sensitivity to appetitive cues (BAS) were more likely to experience positive emotions and events in everyday life than highly BIS sensitivity participants. Eriksson et al. [44] found that higher levels of BIS were associated with poorer inhibitory control and decreased accuracy in the modified stop-signal task in a non-athlete sample. A recent study found that the relationship between a BAS and positive affect could be mediated via emotional intelligence [45].

### 1.3. Self-Efficacy and BAS Associations

Self-efficacy and BAS sensitivity are positively related to each other and to better performance [46,47,48,49]. Research indicates that self-efficacy is associated positively with BAS and sensitivity to reward [47]. In addition, heightened BAS individuals and those with high self-efficacy tended to maintain initial motivation following negative performance, whereas people with high BIS and sensitivity to punishment were strongly demotivated in that condition.

A recent study [48] indicated that adolescents who scored high in BAS and self-efficacy demonstrated great enjoyment during high-intensity exercises. These positive emotions can maintain the systematic training that leads to high achievements in sports performance. The study on vocational interests among U.S. college students found a positive relationship of BAS with social and enterprising self-efficacy [46]. Other research regarding dental behavior (i.e., flossing) among undergraduate psychology students found an indirect effect of self-efficacy on the relationship between approach motivational orientation and health-related change considered as a goal to achieve health behavior [49].

### 1.4. Research Objectives and Hypotheses

Although associations of self-efficacy with BAS, self-efficacy with sports achievements, or BAS with sports performance were found in previous studies, to date, the association between predisposition to sport success, self-efficacy, and behavioral approach motivational system were never examined in one sample of athletes. Self-efficacy plays a crucial role in the professional sports domain. As it was previously established [26], self-efficacy moderates the association between competitive anxiety and sports performance, and can decrease stress and negative emotions during competitions, helping control the situation and managing coping strategies to increase successful performance. Self-efficacy contributes to decision-making and the choice of goals and affects the level of effort and motivational engagement in solving difficult problems [1,2].

According to social cognitive theory [2], human activity is determined by the reciprocal associations between personal dispositions (such as personality traits, attitudes, perceptions, and beliefs), behavioral factors (e.g., self-regulation, physical activity, and sports performance), and environment (e.g., specific social interaction and the situation during a sports competition).

Self-efficacy was found as a mediator in the relationships between the Big-Five personality traits and academic performance [50,51] and between a proactive personality and creative performance [52] and between goal setting and performance in the context of human resource management [53]. Furthermore, Sherman et al. [49] found the mediating effect of flossing self-efficacy on the relationship between BIS/BAS motivational system, flossing intention, and health-related flossing behavior. However, the mediating role of self-efficacy between BAS and sports performance had not yet been explored.

Explaining the mechanism responsible for sports success is crucial for athletes, coaches, and sports administrators to plan the appropriate mental training, improve athletic skills, and achieve successful sports performance. The examination of specific correlations between relatively stable temperament traits, a general sense of self-efficacy and predispositions to sport success may also contribute to better selection and qualification of athletes for specific sports disciplines. Particularly, we are interested in exploring whether BAS, self-efficacy, and predisposition to sport success characterize exclusively elite athletes in speed skating (EASS). Speed skating requires perfect balance and coordination skills as well as adequate muscle strength that improves over years of performing specific aerobic exercises.

We define “elite athletes” as those at the highest standard of performance, regularly competing at the highest level of a given sports discipline (both national and international level), and selected to represent the nation [54]. The highest level of competition required an athlete a special psychological preparation to effectively cope with stress and unexpected environmental factors. The national team of elite speed skating athletes participates in mental training, which considers the specific needs of the individual during sports training and the extremely difficult situations at competition.

Finding the psychological factors responsible for success in sports at the highest level of sports competition may be helpful in the future in the preparation of an appropriate mental training plan by a sports psychologist, taking into account individual differences in the athlete’s traits, psychosocial characteristics, and sport predispositions. Knowledge of the specific configuration of the variables contributing to sports success is essential for its use by athletes, coaches, and sports psychologists. To find specific characteristics of the EASS, this sample is compared in this study with athletes representing other sports disciplines, i.e., physical education students (PES).

This study explore the relationship between the approach motivational system, self-efficacy, and perceived sports success in athletes, controlling for gender, sports discipline, and sport level. Based on previous research, we hypothesize that:

**H1.**  *Women will score lower than men in self-efficacy [18,19,25,26,27,28,29,30,31] and predisposition to sport success [55] but not differently from males in BAS.*

**H2.**  *The EASS sample will score higher than PES in BAS, sports success, and self-efficacy, consistent with previous studies [13,15,17].*

**H3.**  *Athletes at higher levels of sports competition will score higher in BAS, self-efficacy, and predisposition to sports success than those at lower levels [11,12,13,14,15,16,17,18,19,20,21,22,23,42].*

**H4.**  *Self-efficacy will play a mediating role in the relationship between BAS and predisposition to sports success, taking into account previously established associations between these variables [2,36,37,38,39,40,41,42,43,44,45,46,47,48,49] and research on the mediating role of self-efficacy in the fields of academic achievement, management, and healthy behavior [50,51,52,53].*

## 2. Materials and Methods

### 2.1. Study Design and Participants

The cross-sectional study was conducted online from 3 August to 30 November 2020, in Poland. The online survey was presented via Google forms. Invitation to the study was disseminated by e-mail to elite athletes in speed skating (EASS) and physical education students (PES). Informed consent was presented at the first website, and only those who agreed participated in the study. The eligibility criteria for participation in the study depended on the sample. The criterion for inclusion in the sample EASS was to be a member of the NSST in Poland and to be at least 16 years old. All members of the National Speed Skating Team (NSST) were invited to the study in 2020.

The physical education students (PES) were recruited from the Faculty of Physical Education and Physiotherapy at Opole University of Technology, Poland. The criterion for inclusion in the second sample was the age of 16 and to be a physical education student at PA classes. In Poland, PES are preparing for the profession of physical education (PE) teacher in primary and secondary schools and for future work as a trainer and/or instructor in a selected individual or team sport discipline. The bachelor’s study in PE lasts three years, whereas the master lasts two more years and gives more competencies.

Polish PES complete 25 academic hours weekly each of two semesters at year (average semester last 15 weeks), including 40% of practical training in one of two specializations among swimming, athletics, team sports (football, handball, volleyball, and basketball), and individual sports (e.g., fitness, self-defense, aerobics, table tennis, and strength exercises) In addition, one summer camp of 60 h (with swimming, sailing, and windsurfing) and one winter camp (45 h of skiing, downhill skiing, and snowboarding) must be completed among undergraduates. PES can be members of the Academic Sports Association, participating in additional sports training competitions in a selected sports discipline.

The Scientific Research Ethics Committee of the University of Opole (Poland) approved the study methodology (decision No. 6/2020, 13 July 2020). In addition, the study among EASS was approved by the President and Sports Director of the Polish Speed Skating Association and coach, sports doctor, and sports psychologist of the National Speed Skating Team (NSST) in Poland.

### 2.2. Measures

#### 2.2.1. Sport Performance

The Sports Success Scale (SSS) was developed by Mousavi and Vaez Mousavi [55] based on related instruments and theoretical foundations (including the flow state model, motor learning models, and performance motivation theory), as well as expert opinions. The SSS is a self-report tool that consists of 29 items in six subscales: Flow state (five items; e.g., “Training is interesting and enjoyable for me”), Attention (five items; e.g., “When I’m playing, usually nothing distracts me”), Technique (four items; e.g., “The speed of my technique is appropriate”), Sensitivity to error (five items; e.g., “In performing the skill, I’m very careful”), Commitment (five items; e.g., “I respect my sport and its rules”), and Achievement (five items; e.g., “Recently, in most competitions, I have been encouraged by my coach”).

Participants rate each item on a six-point Likert-type scale (1 = “Strongly disagree”, 6 = “Strongly agree”). The total SSS ranges from 29 to 174, and higher scores indicate a better personal predisposition to sports achievements. The SSS was found to be a reliable instrument regarding internal reliability assessed by Cronbach’s α = 0.89 for the total score, and 0.89, 0.88, 0.89, 0.88, 0.89, and 0.89 for Flow state, Attention, Technique, Sensitivity to error, Commitment, and Achievement, respectively [46]. The test–retest reliability with 21 days intervals was 0.90.

The SSS was translated into Polish by a bilingual expert, and then back-translated into English, following the guideline of Beaton et al. [56]. In the present study, Cronbach’s α was 0.89 for the total score, and 0.61, 0.73, 0.62, 0.61, 0.67, and 0.65 for Flow state, Attention, Technique, Sensitivity to error, Commitment, and Achievement, respectively. Due to the low reliability of the subscales and the lack of Polish validation of the tool, only the the total SSS will be analyzed in subsequent statistical tests.

#### 2.2.2. Self-Efficacy

The General Self-Efficacy Scale (GSES) was developed by Schwarzer and Jerusalem [57] to measure perceived confidence in one’s ability to adapt, cope and succeed in specific situations in both daily activities and stressful events. The GSES is a self-report and unidimensional measure, consisting of 10 items (e.g., “I can always manage to solve difficult problems if I try hard enough”). The total score ranges from 10 to 40, with higher scores interpreting a stronger belief that one’s actions are responsible for successful outcomes.

The response to each item can be selected among four options assessing truthfulness in relation to oneself, using a four-point Likert scale (from 1 = “Not at all true” to 4 = “Completely true”). Research indicates that people who scored higher in self-efficacy are more likely to attempt a particular behavior. The internal consistency (Cronbach’s α) of GSES ranged from 0.76 to 0.90 in the original study [57] and from 085 to 0.90 in the Polish samples [58,59]. In the present study, Cronbach’s α was 0.88.

#### 2.2.3. Reinforcement Sensitivity

The Reinforcement Sensitivity Questionnaire (RSQ) was created by Smederevac et al. [60] as a 29-item self-report measure of five dimensions of the revised Reinforcement Sensitivity Theory, namely Behavioral Approach System (BAS, six items; e.g., “I readily accept new and exciting situations”), Behavioral Inhibition System (BIS, seven items; e.g., “I often worry that I may be criticized”), and three scales of Fight-Flight-Freeze Systems (FFFS): Fight (six items; e.g., “Whenever someone hurts me, I immediately fight back”), Flight (five items; e.g., “If I happen to be around aggressive people, I try to get away”), and Freeze (five items; e.g., “Even the presence of some people or things paralyzes me totally”).

Participants respond to each item using a 4-point Likert scale (from 1 = “I completely disagree” to 4 = “I completely agree”). The reliability scores assessed in previous research using Cronbach’s α were 0.65, 0.64, 0.74, 0.70, and 0.78, for the BIS, BAS, Fight, Flight, and Freeze subscales, respectively [49]. The test–retest and split-half suggested suitable reliability for each scale of the RSQ among patients who suffered from chronic pain [61]. We translated the RSQ into Polish using a bilingual expert, and then back-translated it into English, as recommended by Beaton et al. [56]. In the present study, Cronbach’s α values were 0.75, 0.57, 0.69, 0.32, 0.82, and 0.76 for BIS, BAS, Fight, Flight, Freeze, and total FFFS, respectively. The mean BAS item-scale correlation was *r* = 0.31.

#### 2.2.4. Demographics

The demographic data were collected regarding age, gender, training internship (years), sports discipline (speed skating, other disciplines), and sport level of competitions (local, regional, national, or international). In addition, PE students were asked about their study faculty, study year, and study level (undergraduate or graduate), whereas EA was requested to select sports category (Junior, Youth, or Senior), and sport class (Master International, Master, First, Second, or Third).

Participants in the study were 156 athletes, including 91 (58.33%) men and 65 (41.67%) women, aged 16–34 years (*M* = 21.57, *SD* = 3.58). The total sample of athletes consisted of two groups: elite athletes in speed skating (EASS) who were members of NSST in Poland, and physical education students (PES) at the Opole University of Technology in Opole, Poland.

In the EASS sample (*n* = 54), 29 were men and 25 women, aged between 16 and 34 years (*M* = 19.26, *SD* = 4.08). Among EASS, 15 people competed for a sprint on short-track, 15 sprint on long-track, and 23 intermediate runs and all-around-event on long-track. The mean training internship in the EASS sample was 9 years (*M* = 9.11, *SD* = 4.36, ranging from 1 to 22 years). EASS sample was represented by a Junior (*n* = 28), Youth (*n* = 11), and Senior (*n* = 15) categories. In the Master International 13 individuals, the Master class 18 people, the First sports class was 14 athletes, and the Second class represented nine individuals. Participants competed at the National (*n* = 17) and International (*n* = 37) levels. EASS individuals are engaged in four to seven training sessions a week (2–4 h each time) and participated in competitions almost every weekend during the sports season (from October to March).

Among PES (*n* = 102), 62 were men and 40 women, with a mean age of 23 years (*M* = 22.79, *SD* = 32.57, ranging from 20 to 34 years). The average training period in the PES sample was 9 years (*M* = 9.00, *SD* = 4.86, ranged from 0 to 21 years). Among the PE students, 69 were undergraduate (Bachelor’s degree) and 35 graduate (Master of Science degree), including 9 people in the first year, 79 in the second, and 15 in the third study year.

PES represented sport disciplines (from amateur to professional levels), including football (*n* = 31), volleyball (*n* = 10), athletics (*n* = 9), basketball (*n* = 8), handball (*n* = 8), combat sports and martial arts (*n* = 8), fitness (*n* = 7), bodybuilding (*n* = 4), swimming (*n* = 4), dancing (*n* = 4), badminton (*n* = 2), cross fit (*n* = 2), gymnastic (*n* = 2), table tennis (*n* = 2), and cycling (*n* = 1). PES competed at Local (*n* = 22), Regional (*n* = 34), National (*n* = 29), and International (*n* = 17) levels. PES reported to participate in trainings from 1 to 7 days a week (*M* = 4.09, *SD* = 1.6), and average time of training duration ranging between 1–3 h (*M* = 2.14, *SD* = 0.49).

### 2.3. Statistical Analysis

A priori analysis was conducted using G*Power 3.1. software to calculate the expected sample size for planned statistical tests. To detect an effect size of Cohen’s *d* = 0.50 with 80% power (α = 0.05, one-tailed), the G*power software [62] suggests we would need 51 participants per group (*N* = 100) for an independent samples Student’s *t*-test (non-centrality parameter δ = 2.52, critical *t* = 1.66). For correlation analysis, with effect size ρ = 0.30 and 80% power (α = 0.05, two-tailed), we expected sample size of 84 people (critical *r* = −0.22, 0.22). The model’s minimal sample size in a multiple regression analysis with 9 predictor variables should include 56 participants, considering effect size ρ = 0.30 and 80% power (α = 0.05, two-tailed). The present sample size was 156, which can increase the power of the statistical analysis, which was found a-posteriori as 0.93 for Student’s *t*-test, 0.89 for one-way ANOVA, 0.96 for correlations, and 0.99 for multiple regression analysis.

No questionnaire had missing data because the Google forms were insured against incomplete data. Student’s *t*-test was performed to examine differences between genders, and between EASS and PES groups in BAS, self-efficacy, and self-reported predispositions to sports success. A one-way ANOVA was used to examine differences between athletes representing three competition levels (Local/Regional, National, and International) in BAS, sports success, and self-efficacy. The effect size was assessed using Cohen’s *d* (with cut-off 0.20, 0.50, and 0.80 for small, medium, and large effect, respectively) for Student’s *t*-test, and using η^2^*_p_* (with cut-off values of 0.01, 0.06, and 0.14 for small, medium, and large effects, respectively) for the ANOVA results [63]. Association of sports success with self-efficacy and BAS was assessed using Pearson’s correlations.

Hierarchical regression analysis was performed for the total score of sports success as an explained variable and other variables (BAS and self-efficacy). Demographic variables, such as gender (coded 0 = Women, 1 = Men), age (continuous variable), sport discipline (coded 0 = PES, 1 = EASS), and sport level (Local/Regional = 1, National = 2, International = 3) were included in the first step, five scales of temperament in the second step (BAS), and self-efficacy in the third step of regression analysis. The multiple linear regression was conducted, with an enter method of introducing independent variables into the model.

**Figure 1:**
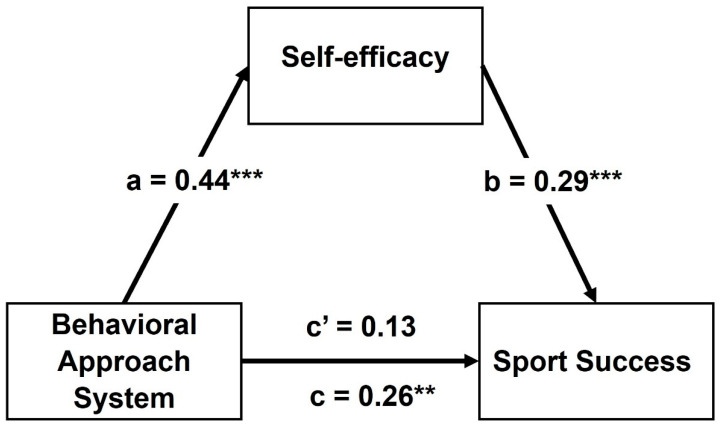
Simple mediation model of association between Behavioral Approach System (BAS) and Sport Success via self-efficacy in the total sample (*N* = 154), by controlling of age, gender, and group (Elite Athletes and Physical Education students); *R*^2^ = 0.16, *F*(5, 150) = 5.87, *p* < 0.001. Regression effects are standardized β coefficients. ** *p* < 0.01, *** *p* < 0.001. The total effect of BAS on Sport Success is β = 0.26, *p* < 0.01. The direct effect of BAS on Sport Success, controlling for self-efficacy as a mediator, is statistically insignificant β = 0.13.

Finally, the mediating effect of self-efficacy on the relationship between BAS and sports success was examined using Model 4 in PROCESS v3.5. Macro for SPSS (IBM Polska Sp. z o.o., Warszawa, Poland) [64]. Model 4 is appropriate for simple mediation analysis, with one mediator variable (Figure 1). Three demographic variables (gender, age, and sports level) were included in the model as potential confounders and sources of bias. In addition, Model 5 of PROCESS was applied to test moderated mediation, with sport discipline as a moderator variable in the association between BAS and sports success. All analyses, including Student’s *t*-test, Pearson’s correlation, linear regression, and the conditional effect in mediation and moderated mediation analysis, were examined based on a bootstrapping procedure with 1000 samples. A bootstrap confidence interval of 95% *CI* is interpreted as a significant effect if not included “0”. All statistical analyses were performed using IBM SPSS ver. 26 software.

## 3. Results

### 3.1. Descriptive Statistics

Initially, 161 people responded to the invitation; however, two EASS and three PES individuals refused to participate in the study. The final sample consisted of 156 individuals, including 65 women (41.7%) and 91 men (58.3%). The mean age of athletes was 22 (*M* = 21.60, *SD* = 3.52). Descriptive statistics are shown in Table 1, including a range of scores, mean (*M*), standard deviation (*SD*), skewness, and kurtosis. If skewness and kurtosis range between +2 and −2, the data can be considered as having acceptable properties for parametric statistics [65]. This criterion was met for BAS and GSES; however, kurtosis for SSS (4.94) was not satisfactory; therefore, the resampling procedure using Bootstrapping technique with 1000 replicates was implemented for all statistical methods. Bootstrapping (BS) is often performed if the sample size is small and the distribution does not meet normal expectations. Analysis of correlation (Table 1) indicates that BAS is associated positively with predispositions to sport success (BS 95% *CI* = 0.095, 0.394) and self-efficacy (BS 95% *CI* = 0. 231, 0.506), and predispositions to sport success are related positively to self-efficacy (BS 95% *CI* = 0.307, 0.582).

### 3.2. Group Differences

The Student’s *t*-test was used to examine differences between genders (H1) and between elite athletes (EASS) and physical education students (PES) groups (H2) in BAS, predisposition to sport success, and self-efficacy. Women (*n_W_* = 65) did not differed from men (*n_M_* = 91) in BAS (*M_W_* = 2.74, *SD_W_* = 0.40; *M_M_* = 2.84, *SD_M_* = 0.40; *t*(154) = 1.61, *p* = 0.109, Cohen’s *d* = 0.26, BS 95% *CI* = −0.230, 0.025), predispositions to sport success (*M_W_* = 126.20, *SD_W_* = 12.19; *M_M_* = 128.40, *SD_M_* = 19.79; *t*(154) = 0.79, *p* = 0.429, Cohen’s *d* = 0.13, BS 95% *CI* = −7.097, 2.971), and self-efficacy (*M_W_* = 31.35, *SD_W_* = 4.96; *M_M_* = 32.32, *SD_M_* = 4.152; *t*(154) = 1.32, *p* = 0.189, Cohen’s *d* = 0.22, BS 95% *CI* = −2.454, 0.520).

The EASS sample (*n*_EASS_ = 54, *M_EASS_* = 2.61, *SD_EASS_* = 0.39) showed significantly lower scores in BAS than PES group (*n*_PES_ = 102, *M_PES_* = 2.89, *SD_PES_* = 0.38), *t*(154) = −4.37, *p* < 0.001, Cohen’s *d* = 0.74, BS 95% *CI* = 0.156, 0.409. There were no differences between the EASS and PES groups in predisposition to sport success (*M_EASS_* = 128.87, *SD_EASS_* = 15.20; *M_PES_* = 126.75, *SD_PES_* = 17.95; *t*(154) = 0.74, *p* = 0.460, Cohen’s *d* = 0.13, BS 95% *CI* = −7.450, 3.188), and self-efficacy (*M_EASS_* = 31.61, *SD_EASS_* = 5.06; *M_PES_* = 32.08, *SD_PES_* = 4.22; *t*(154) = −0.61, *p* = 0.541, Cohen’s *d* = 0.10, BS 95% *CI* = −1.091, 2.046).

A one-way ANOVA was performed, testing H3 about differences in BAS, predispositions to sport success, and general self-efficacy, dependent on level of sport competitions: Local/Regional (*n*_L/R_ = 56), National (*n*_N_ = 46), and International (*n*_I_ = 54). No differences were found in BAS (*M*_L/R_ = 2.85, *SD*_L/R_ = 0.37; *M*_N_ = 2.76, *SD*_N_ = 0.46; *M*_I_ = 2.77, *SD*_I_ = 0.39; *F*(2, 153) = 0.89, *p* = 0.412, η^2^*_p_* = 0.01), Sport Success Scale (*M*_L/R_ = 124.55, *SD*_L/R_ = 15.30; *M*_N_ = 127.83, *SD*_N_ = 20.43; *M*_I_ = 130.22, *SD*_I_ = 15.29; *F*(2, 153) = 1.55, *p* = 0.216, η^2^*_p_* = 0.02), and general self-efficacy (*M*_L/R_ = 31.89, *SD*_L/R_ = 3.97; *M*_N_ = 31.54, *SD*_N_ = 4.69; *M*_I_ = 32.26, *SD*_I_ = 4.95; *F*(2, 153) = 0.31, *p* = 0.734, η^2^*_p_* < 0.01). The BS 95% *CI* confirmed no group differences for each variable.

### 3.3. Predictors of Sports Success

The hierarchical regression analysis was conducted in a total sample of athletes (*N* = 156) to examine significant predictors of predispositions to sports success among BAS temperamental trait and self-efficacy. Some demographic variables, such as gender, age, sports discipline (speed skating vs. other sport disciplines), and sport level (local or regional, national, and international), were included in the model in the first step as potential confounders. The results are shown in Table 2. Demographic variables explain 5% of sports success variance, and only older age (BS 95% *CI* = 0.091, 1.676) was related to higher sports success.

However, when temperamental traits were included in the regression model in the second step of the analysis, age was no longer significantly related to the dependent variable (BS 95% *CI* = 0.013, 1.528), and solely BAS was found to be a significant predictor of sports success (BS 95% *CI* = 2.772, 17.191). An explained variance increased to 10% in comparison to the first model. When self-efficacy was added to the model in the third step of regression analysis, neither age (BS 95% *CI* = −0.219, 1.222) nor BAS (BS 95% *CI* = −1.308, 11.553) was longer found to be a significant predictor of sports success; however, solely self-efficacy was positively related (BS 95% *CI* = 0.336, 1.908). The last model explained 17% of the total sports success variability.

Since the association between BAS and sports success decreased when self-efficacy was included in the regression model, it is reasonable to expect that self-efficacy plays a mediator role in these relationships. We performed a test of feasibility mediation analysis, following Murphy’s [66] recommendation. The first assumption was fulfilled, since both *r*_xm_ = 0.43 and *r*_my_ = 0.36 are larger than *r*_xy_ = 0.23. The second criterium was met, since *r*_xm_ and *r*_my_ are closer in value to $\sqrt{r_{\mathrm{xy}}}$ = 0.48 than to the value of *r*_xy_. Therefore, the mediation hypothesis will be examined in the next section.

### 3.4. Mediating Effect of Self-Efficacy on the Relationship between Behavioral Approach System and Sport Success

Finally, we examined the mediating role of self-efficacy in the relationship between BAS and sports success. The Bootstrapping method based on 1000 replications and 95% *CI* were used to Demographic variables, such as age, gender, and group (EASS and PES), were included in the mediation model as confounders. The results of the mediation analysis are shown in Figure 1.

Self-efficacy can be predicted by BAS, β = 0.44, *b* = 4.87, *SE b* = 0.86, *t* = 5.65, *p* < 0.001, BS 95% *CI* = (3.39, 6.36). The BAS explains 22% of the self-efficacy variance, *R*^2^ = 0.22, *F*(4, 151) = 10.79, *p* < 0.001. The BAS is a predictor of sport success directly. Total effect was significant, β = 0.26, *b* = 10.85, *SE b* = 3.48, *t* = 3.12, *p* < 0.01, BS 95% *CI* = (3.96, 17.73), explaining 10% of sport success variance, *R*^2^ = 0.10, *F*(4, 151) = 4.04, *p* < 0.01. When self-efficacy was included as a mediator to the model of association between BAS and sport success, the explained variance increased to 16%, *R*^2^ = 0.16, *F*(5, 150) = 5.87, *p* < 0.001, while association between BAS and sport success dropped to an insignificant level (direct effect), β = 0.13, *b* = 5.49, *SE b* = 3.70, *t* = 1.48, *p* = 0.14, BS 95% *CI* = (−0.80, 11.35). Self efficacy was significant predictor of sport success, β = 0.29, *b* = 1.10, *SE b* = 0.32, *t* = 3.47, *p* < 0.001, BS 95% *CI* = (0.34, 1.87). This means that self-efficacy completely mediates the relationship between BAS and sport success; indirect effect of BAS on sport success via self-efficacy was 5.36, BS *SE* = 2.17, 95% *CI* = (1.51, 9.99), completely standardized indirect effect.

**Figure 2:**
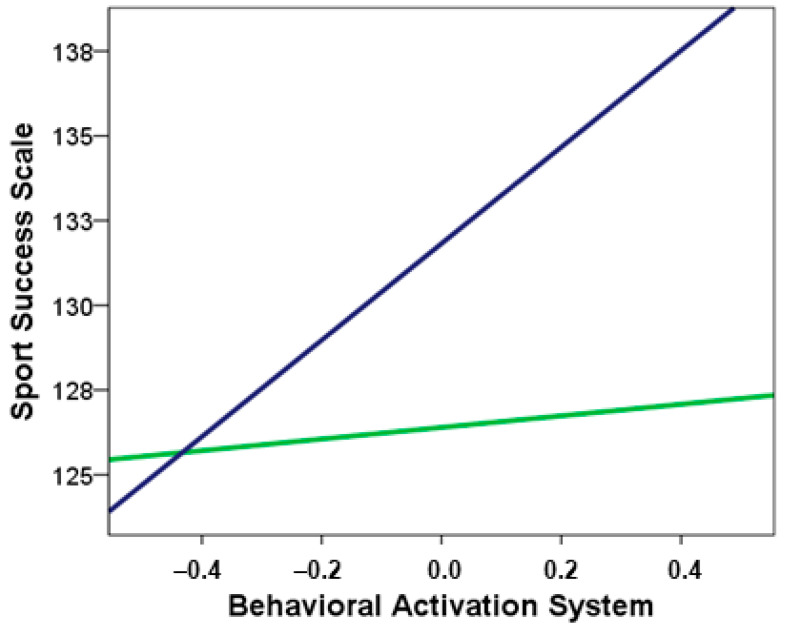
Interaction effect between BAS and sport discipline (speed skating vs. other sports) on predispositions to sport success. The navy-blue line represents speed skating (EASS sample) and the green line indicates other sports (PES group).

The moderated mediation analysis was conducted in the next step, to examine whether the interaction between discipline (speed skating vs. other disciplines) and BAS is presented in the mediation model. The results indicates that sport discipline has marginal effect on sport success, but significant if taking into account bootstrapping analysis, *b* = 5.43, *SE b* = 2.88, *t* = 1.88, *p* = 0.062, BS 95% *CI* = (0.392, 10.505). Similarly, interaction effect between sport discipline and BAS on sport success was marginally confirmed, *b* = 12.53, *SE b* = 96.94, *t* = 1.81, *p* = 0.073, BS 95% *CI* = (0.743, 25.013). As it is shown in Figure 2, the regression slope is much higher for the EASS sample than for the PES group, which means that the association between BAS and sports success is stronger in speed skating than for other sports disciplines. The complete moderated mediation analysis explains 17% of sport success variance, *R*^2^ = 0.17, *F*(4, 151) = 7.70, *p* < 0.001 was 0.13, BS *SE* = 0.06, BS 95% *CI* = (0.03, 0.26).

## 4. Discussion

### 4.1. Differences between Athletes Regards to Gender, Sports Discipline, and Sports Competition Level

Contrary to hypothesis H1, gender differences were not found in this study. Previous studies showed that men scored higher in self-efficacy [18,19,25,26,27,28,29,30,31] and predisposition to sport success [55], which was determined by the perception of gender stereotypes [24,25,30,31]. However, speed skating is a relatively new sport and may not be associated with any stereotypical perception. For example, among elite artistic roller skaters, gender differences in self-efficacy were also not presented [13]. Furthermore, in recent decades, women have also achieved excellent athletic performance, so they may feel less reliant on the concept of gender in athletic activity.

The structure of societies is changing under the influence of civilization changes and the idea of sustainable development in democratic countries, which may contribute to overall less stereotypical perception by young generations. The H2 was not confirmed, since the EASS sample showed significantly lower scores in BAS than PES, with no differences in self-efficacy, and the total SSS. High BAS may lead to high impulsivity and addictive behaviors [67], which can worsen sports performance. Therefore, lower scores in BAS in EASS individuals, compared to PES students, can be more adaptable, decreasing the risk of addiction and promoting more controllable behavior during competitions as extreme stressful events.

The EASS includes the best athletes in speed skating in the country. This strict selection can only include those athletes who successfully adapt to systematic training under the tough environment and extreme competitive conditions of which lower BAS levels can be helpful. Unfortunately, studies on BIS-BAS motivational systems are very scarce in the field of sport and physical activity, so more research is necessary to fully explain these questions in the future.

On the other hand, most variables did not differentiate EASS from the PES sample. No differences between groups in self-efficacy, inconsistent with previous research [13,15,17]. Although the level of sports competition is higher on average in EASS than PES group in the present study, a significant number of PES competed at National and International levels. The average time of training and its duration were similar in both groups EASS and PES. These similarities between PA engagement may explain the lack of significant differences in self-efficacy and predispositions to sport success. However, a distinct association pattern between these variables was found in EASS and PES samples, which will be described in the next section.

In contrast to H3 and previous studies [11,12,13,14,15,16,17,18,19,20,21,22,23,42], the differences in BAS, self-efficacy, and predisposition to sports success were not found in athletes regarding levels of sports competition. BAS refers to an incentive system and can characterize athletes in general, regardless of their athletic level. Future research should examine whether differences in BAS are presented between athletes and the non-athlete population. Furthermore, psychological predisposition to sport success may be similar across athletes at various levels of competition; however, success in particular sports disciplines may be more related to systematic training, unique techniques, specific motoric skills, and sports talent. The self-efficacy was measured in this study in relation to the general level presented in life, independent of specific sports situations. The following study should use self-efficacy related to sports context.

### 4.2. Association between Self-Efficacy, BAS, and Sports Success

The present study examined associations between self-efficacy, BAS temperamental trait related to basic motivational systems (consistent with the Reinforcement Sensitivity Theory), and individual predispositions to sports performance among athletes. The positive relationship between sports success and self-efficacy was confirmed in the study. The results of this study are consistent with previous research performed among athletes [11,12,13,14,15,16,17,18,19,20,21,22,23], as well as in the domains of physical activity [5,6,7,8,9,10,11] and academic achievements [3,4]. According to theory [1,2], people with a high sense of self-efficacy do not give up due to difficulties, treat such situations as an opportunity to master the task, and believe in learning from failures [68].

In contrast, people with low self-efficacy are not motivated to resolve challenging tasks and show avoiding tendency, particularly after failure [2,69,70], which can explain the association between self-efficacy and sports success in this research We found a moderate correlation between self-efficacy and sports success in the total sample. The results are consistent with previous meta-analyses [11]. Furthermore, the present study showed that self-efficacy explained solely 6% variance of the total SSS. Among an international sample of 40 elite athletes, ski jumping performance was moderately related to self-efficacy and explained approximately 14% of the overall World Cup ski jumping [21]. The differences may result from different performance measurements. In the present study sport, success predispositions were assessed using a self-report survey, while Sklett et al. [21] have used objective measures of performance during a competition.

BAS was related positively to the total SSS scores in the total sample of athletes. Hierarchical regression analysis showed in the second step that BAS significantly contributes to sport success total score. BAS is responsible for approach and engagement behavior; therefore, it can explain high performance [35,36]. Previous research demonstrated an essential contribution of BAS in high achievement among non-athletes [37,38]. BAS was also examined in the context of sport and physical activity [39,40,41,42]. The positive relationship between BAS and participation in physical activity was established in previous studies [37,38,39].

### 4.3. Mediating Role of Self-Efficacy in the Association between BAS and Sports Success

A mediating role of self-efficacy on the relationship between BAS and sports success was fully confirmed in this study, consistent with hypothesis H4 and a previous study [2,36,37,38,39,40,41,42,43,44,45,46,47,48,49,50,51,52,53]. According to the RST [32,33,34], an approach and avoidance motivated behavior results from the interaction between neuropsychological personality traits and motivational stimuli that possess a specific value (more or less attractive). Furthermore, beliefs about self-efficacy can also contribute to sports performance [1,2]. Therefore, sports performance can be considered in this study as a goal, eliciting motivational process to achieve, and cognitive assessment of self-abilities to win, and to cope with stressful competition event with success.

The relationship between self-efficacy and BAS was found previously [46,47,48,49]. Sherman et al. [49] argued that self-efficacy plays a vital role in performing a wide range of health behaviors [71]. The presented research is consistent with this assumption, showing the role of self-efficacy in sports performance and approaching temperamental dispositions. In particular, moderated mediation showed that sports success is more dependent on BAS than self-efficacy in elite speed skaters, whereas self-efficacy fully mediates the relationships between BAS and sports success among PES.

The present findings provide preliminary support for the social cognitive theory [1,2] end extend the Reinforcement Sensitivity [32,33,34], by showing how temperamental traits interact with specific sport discipline and cognitive characteristics of athletes, leading to success in the sports domain. Motivational and cognitive systems are related to each other, contributing to either success or bad performance in everyday life, as well as in physical activity and sports competitions. Clearly and Zimmerman highlighted the role of interaction between motivational and cognitive systems in self-regulation processes during deliberate training. Self-reflection and self-evaluation, problem detection, errors correction, and self-rewarding, all played a crucial role during the learning process in athletes with high master orientation.

### 4.4. Limitation of the Study and Directions for Future Research

There is some limitation of this study, which cannot generalize results in a population. The sample size was not large and the sports successes did not meet the assumption of normality, therefore the bootstrapping technique was used for all statistical tests. Future research should include a larger sample of people and be more diverse in terms of sports success (similar numbers of newcomers and experts in sports). The other limitation is that sports experts were represented in one sport discipline, namely elite athletes in a short track speed skating, and compared with a more heterogeneous group of PE students, who represented various disciplines at various levels of competitions. Further research should compare more homogenous and a larger group of athletes representing one discipline but various levels of competitions, with a large group of non-athletes representing the general population.

The cross-sectional design used in these studies limits the cause-effect inference and the predictive power of these results. Further research should be performed longitudinally. All variables used in this study were based on self-report measures, which can limit the obtained results. The RSQ and SSS measures were translated and used for the first time in the Polish samples without specific adaptation and validation studies. This fact was most likely related to the low reliability of subscales in the RSQ and SSS. However, Cronbach’s α value is influenced by a larger number of variables, the number of items within a subscale, and the multidimensional structure of a whole construct [65]. It is also important to note, that the mean BAS item-scale correlation is acceptable (*r* = 0.31). Therefore, the reliability of 0.56 for BAS can be considered poor but sufficient for preliminary analysis [72]. We suggest replicating the study in the future, by using tools with strong validity. Further research could use objective neurophysiological methods to assess BAS, and objective assessment during competitions, to measure sports performance.

## 5. Conclusions

Individual differences in motivational systems related to temperamental approach and avoidance dispositions and self-efficacy contribute to self-report predispositions to sports success. The study found a specific pattern of associations between self-efficacy, approach temperamental disposition, and sports success in elite athletes representing speed skating and among PES representing various sports. Furthermore, we showed the mechanism of how neuropsychological dispositions manifesting in approach behavior affects sports success indirectly by increasing self-efficacy. Coach and sports psychologists can use this study’s results to work on self-efficacy, among highly approach sensitive athletes, to increase their chance for success in sports competitions.

This study demonstrated that the behavioral approach motivational system is moderately associated with self-efficacy among athletes in general and strongly related to predispositions to sport success among EASS. These results may have theoretical and practical contributions to sports psychology. At the theoretical level, the present findings exceed the theory of social cognition and reinforcement sensitivity, showing how basic temperamental traits interplay with cognitive and behavioral factors. At the practical level, the results of this study can be used by athletes, sports instructors, coaches, and sports psychologists, who should apply frequent rewarding during training sessions, inducing positive emotions related to high achievements as much as possible and visualizing appetitive stimuli (e.g., during mental training).

At the same time, increasing self-efficacy is also important for high sports achievements among elite speed skaters but seems yet more essential for athletes representing other sports disciplines. Coaches of collegiate and university teams of athletes may increase self-efficacy in PES, regardless of the level of competition or sports discipline. The training bolstering self-efficacy skills can include goal-setting, facing challenges, accepting failures and criticism positively, avoiding negative emotions (e.g., blaming, shaming, and getting angry), frequently rejoicing (even a small success), remembering and focusing on successes instead of failures, looking beyond short-term losses, increasing self-trust and self-esteem by positive thinking and affirmations, identifying obstacles and thought blocs, and reframing looking at failures by using them as a constructive way to induce positive changes in life and to avoid replicating the same errors in the future.

## Figures and Tables

**Table 1:** Descriptive statistics for the total study sample (*N* = 156).

						Correlations *r*	
Variable	Range	*M*	*SD*	Skewness	Kurtosis	BAS	SSS
Behavioral Activation System (BAS)	1–4	2.80	0.40	−0.23	1.12		
Sport Success Scale (SSS)	35–165	127.48	17.03	−1.16	4.94	0.23 **	
General Self-Efficacy Scale (GSES)	16–40	31.92	4.52	−0.32	0.82	0.43 **	0.36 **

** *p* < 0.01.

**Table 2:** Hierarchical regression for a total score of Sports Success Scale (*N* = 156).

			95% *CI*										
Model	Variable	*b*	*LL*	*UL*	*SE b*	β	*t*	*p*	*R* ^2^	Δ*R*^2^	Δ*F*	Δ*df*	Δ*p*
1	Constant	102.06	81.64	121.73	10.02		10.18	0.00	0.05	0.05	1.96	4, 151	0.104
	Gender	1.60	−3.79	7.09	2.76	0.05	0.58	0.56					
	Age	0.86	0.00	1.75	0.44	0.18	1.96	0.05					
	Sport discipline	2.55	−5.44	10.52	4.05	0.07	0.63	0.53					
	Sport level	2.55	−1.51	6.59	2.05	0.13	1.25	0.21					
2	Constant	75.94	49.81	101.67	13.06		5.81	0.00	0.10	0.05	9.03	1, 150	0.003
	Gender	0.81	−4.47	6.18	2.70	0.02	0.30	0.77					
	Age	0.72	−0.12	1.59	0.43	0.15	1.69	0.09					
	Sport discipline	5.78	−2.31	13.81	4.09	0.16	1.41	0.16					
	Sport level	1.83	−2.15	5.80	2.01	0.09	0.91	0.36					
	BAS	10.66	3.65	17.64	3.54	0.25	3.01	0.00					
3	Constant	62.37	36.01	88.58	13.25		4.71	0.00	0.17	0.06	11.39	1, 149	0.001
	Gender	0.43	−4.69	5.62	2.61	0.01	0.16	0.87					
	Age	0.48	−0.36	1.33	0.42	0.10	1.15	0.25					
	Sport discipline	3.84	−4.07	11.68	3.99	0.11	0.96	0.34					
	Sport level	1.89	−1.95	5.74	1.94	0.09	0.97	0.33					
	BAS	5.33	−2.13	12.77	3.77	0.13	1.41	0.16					
	Self-efficacy	1.08	0.45	1.71	0.32	0.29	3.38	0.00					

Note. BAS = Behavioral Activation System, *CI* = confidence interval, *LL* = lower level, *UL* = upper level.

## Data Availability

The data presented in this study are openly available in Mendeley Data at doi:10.17632/smr3j9hwr4.1 (accessed on 20 January 2022) [73].

## Abbreviations

ANOVA	analysis of variance
BAS	behavioral approach system
BIS	behavioral inhibition system
BS	Bootstrapping
*CI*	confidence interval
EASS	elite athletes in speed skating
FFFS	fight-flight-freeze system
GSES	General Self-Efficacy Scale
IRB	Institutional Research Board
*LL*	lower level
NSST	National Speed Skating Team
PA	physical activity
PE	physical education
PES	physical education students
RSQ	Reinforcement Sensitivity Questionnaire
RST	Reinforcement Sensitivity Theory
SSS	Sports Success Scale
*UL*	upper level
